# TiO_2_/Porous Carbon Composite-Decorated Separators for Lithium/Sulfur Battery

**DOI:** 10.1186/s11671-019-3010-2

**Published:** 2019-05-28

**Authors:** Haisheng Han, Songqiao Niu, Yan Zhao, Taizhe Tan, Yongguang Zhang

**Affiliations:** 1 School of Materials Science and Engineering, Hebei University of Technology, Tianjin, 300130 China; 2 Synergy Innovation Institute of GDUT, Heyuan, Guangdong Province China

**Keywords:** TiO_2_-decorated porous carbon, Modified separator, Lithium/sulfur battery, Polysulfide barrier

## Abstract

The practical application of lithium/sulfur (Li/S) batteries is hindered by the migration of soluble polysulfides (Li_2_S_*n*_, 4 ≤ *n* ≤ 8) from cathode to anode, leading to poor electrochemical stability of the cell. To address this issue, in the present study, a TiO_2_/porous carbon (TiO_2_/PC) composite-coated Celgard 2400 separator was successfully fabricated and used as a polysulfide barrier for the Li/S battery. In TiO_2_/PC, the highly conductive PC with three-dimensional ordered porous structure physically constrains polysulfides and at the same time serves as an additional upper current collector. On the other hand, the TiO_2_ on the surface of PC chemically adsorbed polysulfides during the charge/discharge process. Due to the physical and chemical adsorption properties of TiO_2_/PC composite coating layer, an initial discharge capacity of 926 mAh g^−1^ at 0.1 C and a low fading rate (75% retention after 150 cycles) were achieved. Moreover, in the rate capability test, the discharge capacity for the TiO_2_/PC-modified Li/S battery was recovered to 728 mAh g^−1^ at 0.1 C after high-rate cycling and remained ~ 88% of the initial reversible capacity.

**Electronic supplementary material:**

The online version of this article (10.1186/s11671-019-3010-2) contains supplementary material, which is available to authorized users.

## Background

Among the rechargeable batteries, lithium/sulfur (Li/S) battery has been considered as a promising candidate for the next-generation power supplies because of their high theoretical energy density (2600 Wh kg^−1^) and specific capacity (1675 mAh g^−1^) [1]. Additionally, Li/S batteries also have other advantageous features such as low toxicity, low cost, and high natural abundance [2].

However, there are still some problems hindering the practical application of Li/S batteries. These problems include the following: (i) the insulating nature of elemental sulfur (*σ*_298_ = 5 × 10^−30^ S cm^−1^) would result in the low utilization of the active material; (ii) the volume change resulting from the different volume density of Li_2_S and sulfur leads to a serious capacity decay of the battery; and (iii) the dissolution and diffusion of polysulfides in the electrolyte would cause a low Coulombic efficiency and rapid decline in the capacity [3, 4].

To solve these problems, extensive efforts have been devoted to confine S within the cathode region [5, 6]. A large number of materials such as porous carbon, inorganic oxides, and polymers have been designed and synthesized to trap the polysulfide within the cathodes [7–13]. However, the introduction of high content sulfur-trapping materials inevitably reduces the overall energy densities of the cell. Therefore, various strategies beyond the cathode modification have been explored.

An alternative strategy to suppress the dissolution and diffusion of polysulfides is the modification of the internal structure of the Li/S battery, such as building a coating interlayer on the separator [14, 15]. Thus, different kinds of carbon-based modified separators are widely applied to Li/S batteries to inhibit the diffusion of polysulfides though physical absorption [16, 17]. Li et al. groups reported the reduced graphene oxide/active carbon functional interlayer could improve the cycle performance of Li/S battery [17]. Nevertheless, the weak interaction between the unpolar carbon matrix and polar polysulfides is considered to be insufficient to immobilize the migrating polysulfides. Therefore, carboneous materials are usually composited with the polar metal oxides, such as layered double hydroxide, CeO_2_, which could offer a stronger chemical binding to polysulfides through polar-polar interaction [18–22]. The chemical nature between polysulfides and polar TiO_2_ surface and carbon functional groups has been well-demonstrated both experimentally and theoretically [23, 24].

Herein, we reported a TiO_2_-decorated porous carbon (TiO_2_/PC) as a coating layer on Celgard 2400 separator to suppress the polysulfide shuttle effect. In the TiO_2_/PC composite, TiO_2_ nanoparticles uniformly decorated on the surface of PC could effectively restrain the diffusion of polysulfides by chemical bonding. On the other hand, the PC layer not only ensures the good electrical conductivity of the composite, but also can mitigate the polysulfides dissolution by providing a physical confinement of polysulfides within its porous structure.

## Methods

### Preparation of Li/S Battery with TiO_2_/PC-Modified Separator

#### Preparation of Porous Carbon

**Fig. 1:**
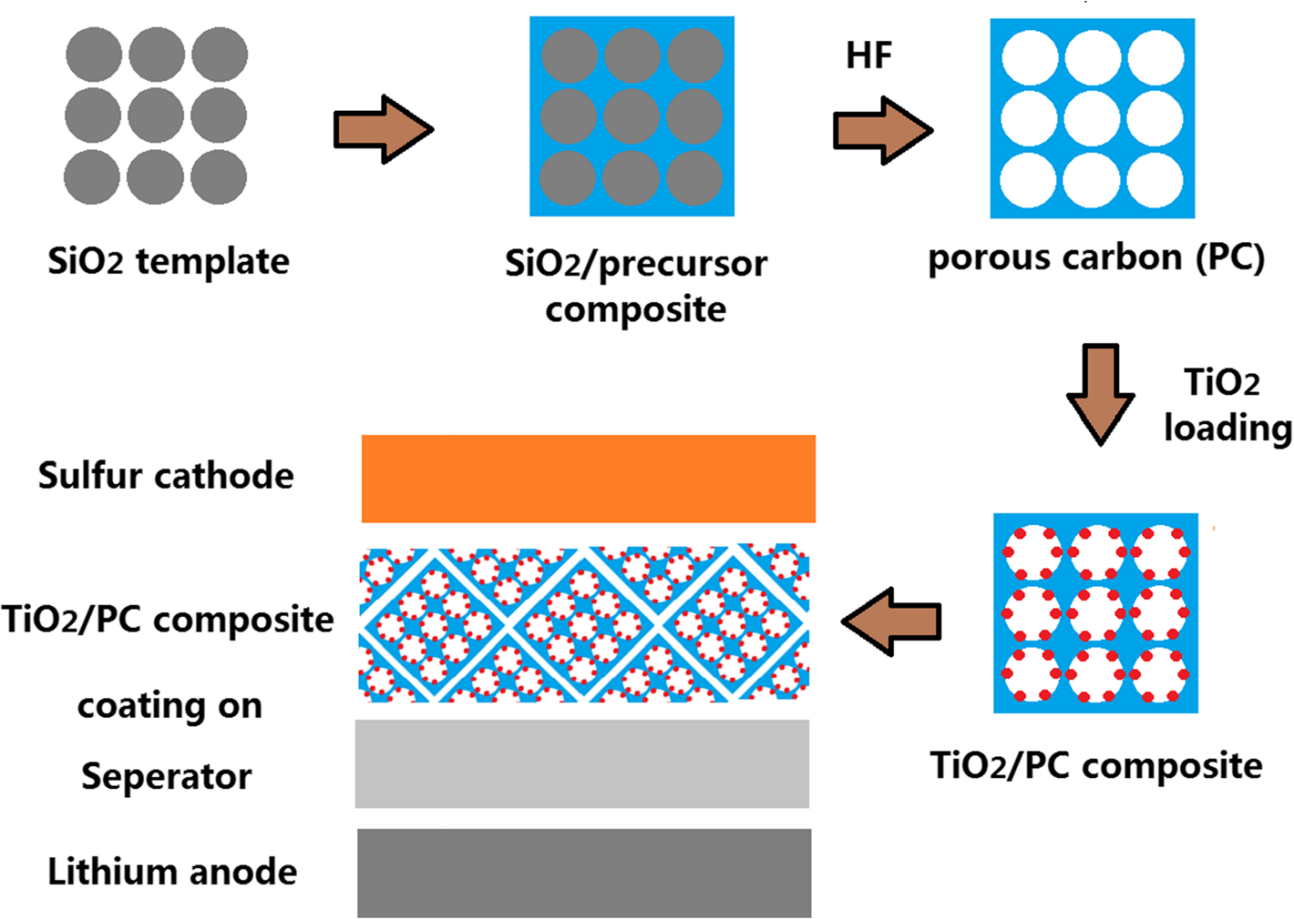
Synthesis of the TiO_2_/PC-modified Celgard 2400 separator for the Li/S battery

Figure 1 displays the schematic representation of the fabrication process of the TiO_2_/PC-modified Celgard 2400 separator. Monodisperse silica microspheres were first prepared by hydrolyzing tetraethyl orthosilicate (TEOS) with an ammonia solution and then centrifugally dispersed in ethanol. The ethanol solution was naturally dried to obtain silica opal, which was then dispersed in a resol solution. Here, resol was used as a carbon source and was treated at 600 °C for 2 h under argon atmosphere with a heating ramp of 2 °C min^−1^ in a tube furnace. An 11% weight loss in the carbonization of resol was observed. Then, the silica opal template was etched by HF solution, and the PC template with ordered porous structure was obtained.

#### Deposition of TiO_2_ on PC

The TiO_2_ presoma solution was prepared by a sol–gel method. First, 2.84 g (0.1 mol) of tetraisopropyl titanate (TTIP), 2.4 g of hydrochloric acid, and 4.0 g of ethylalcohol were mixed and stirred for 1.5 h to form a transparent gel solution. The PC template was soaked in the TiO_2_ solution for 24 h. Then, the PC template deposited with TiO_2_ was collected and naturally dried for 3 days. After that, it was heat treated at 450 °C for 1 h under N_2_ atmosphere for further use.

#### Preparation of the TiO_2_/PC-Modified Separator

A slurry was prepared by mixing 0.7 g TiO_2_/PC, 0.2 g carbon black, and 0.1 g polyvinylidene difluoride (PVDF) in *N*-methyl pyrrolidone (NMP) solvent. The slurry was coated onto the commercial Celgard 2400 separator and dried at 50 °C overnight in a vacuum drying oven. The thickness of TiO_2_/PC on Celgard 2400 separator is 37 μm, and the areal loading of TiO_2_/PC is about 0.5 mg cm^−2^. The TiO_2_/PC-modified Celgard 2400 separator was cut into disks of 1 cm in diameter.

### Material Characterizations

The crystalline structure of the TiO_2_/PC-modified separator was measured by using powder X-ray diffraction (XRD, Smart Lab, Rigaku), with Cu–Kα radiation (*λ* = 1.5406 Å) at the 2*θ* range of 10 to 90°. The morphology of the obtained TiO_2_/PC composite was studied by scanning electron microscopy (SEM, JSM-7100F, JEOL) and transmission electron microscopy (TEM, JEM-2100F, JEOL) with an accelerated voltage of 200 kV (Additional file 1). The contact angle measurement was performed using an JGW-360Y contact angle meter. The functional groups of the TiO_2_/PC-modified separator after charge/discharge were tested by using X-ray photoelectron spectroscopy (XPS, Kratos AXIS Ultra DLD, Al–Kα).

### Electrochemical Measurements

The slurry of the sulfur cathode was prepared by mixing 0.8 g S, 0.1 g carbon black, and 0.1 g PVDF in NMP. The slurry was coated onto Al foil and dried at 60 °C overnight under vacuum condition. The sulfur electrodes were then cut into 1-cm disks. The sulfur loading is approximately 2.0 mg cm^−2^. The amount of electrolyte is around 40 μL. Metallic Li was used as the anode, and the electrolyte used was 1 M LiTFSI in a binary dioxolane (DOL) and dimethoxyethane (DME) solvent (1:1 *v*/*v*). The electrochemical performance was evaluated by coin cells (CR2025) which were assembled in an MBraun glove box under high-purity argon (Ar ≥ 99.9995%). The electrochemical charge/discharge performance was measured between 1.5 and 3 V with a Neware battery tester (BTS-5V5mA) at room temperature.

## Results and Discussion

**Fig. 2:**
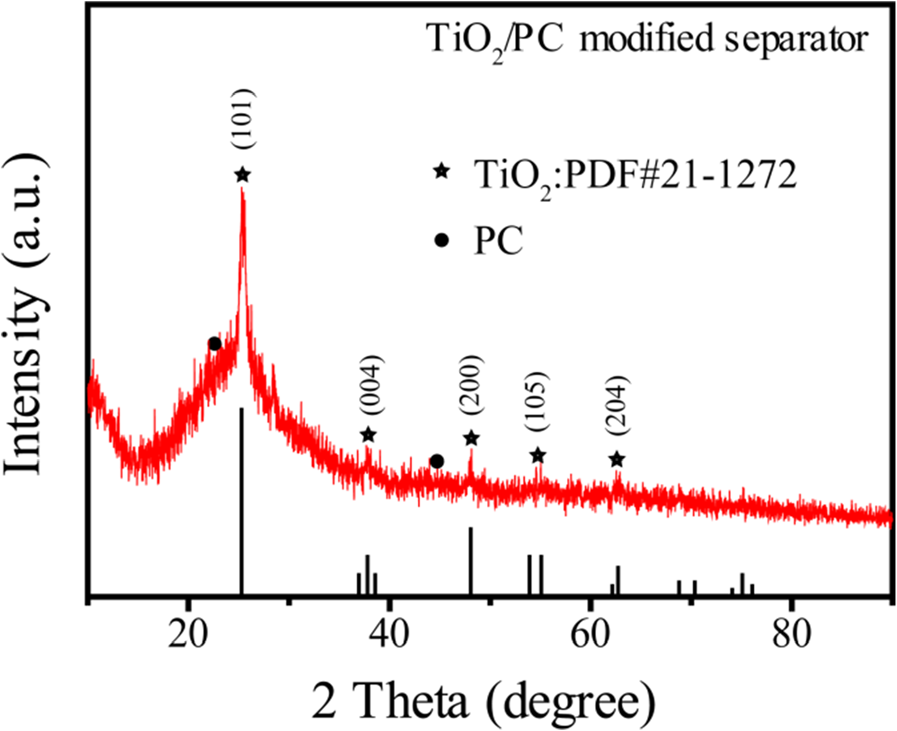
XRD pattern of the TiO_2_/PC-modified separator

Figure 2 shows the XRD pattern for the TiO_2_/PC-modified separator. The crystalline phase was identified as anatase TiO_2_ (JCPDS No.21-1272). Additionally, there were two typical peaks at around 23° and 44°, corresponding to the diffraction from (002) and (100) of carbon, respectively.

**Fig. 3:**
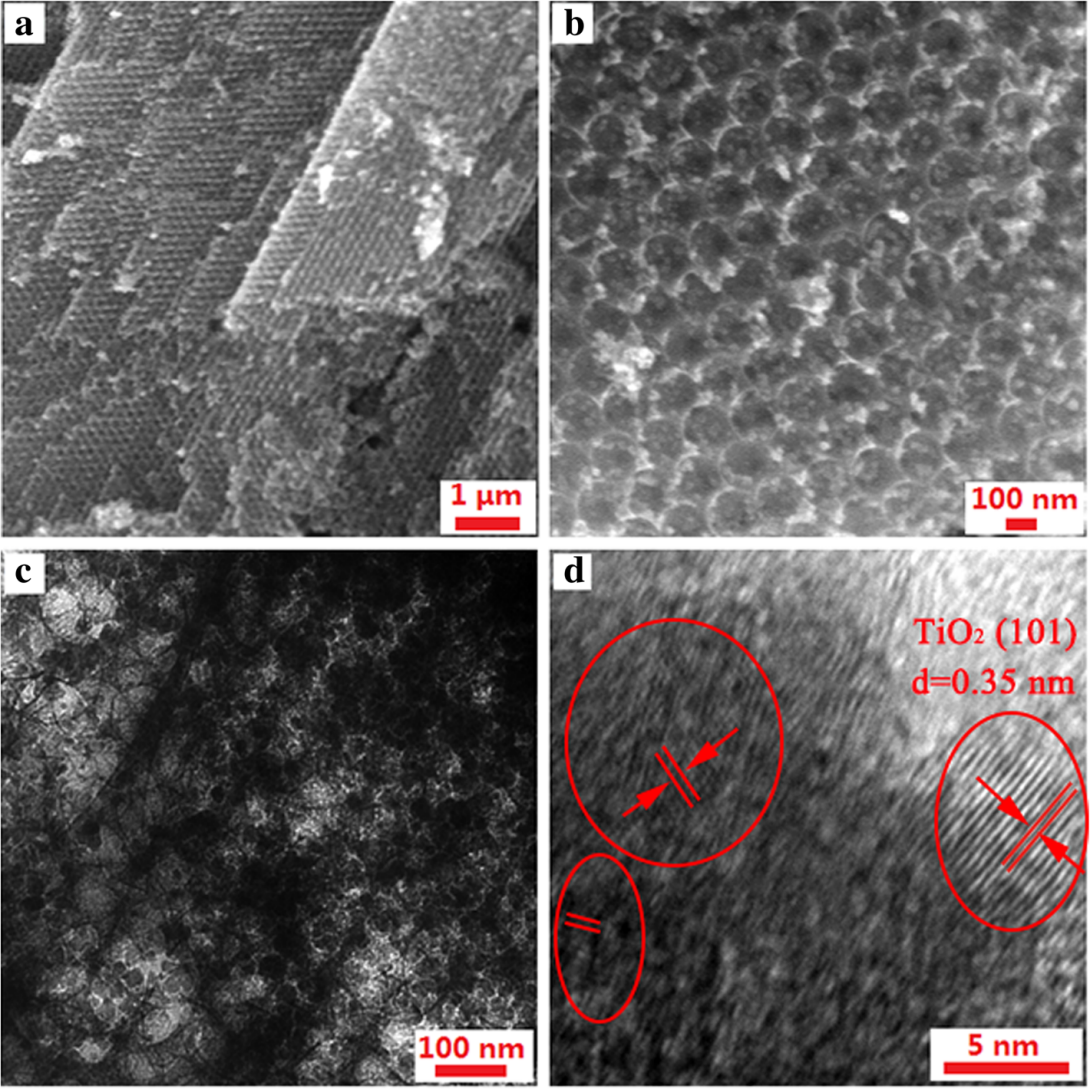
SEM (**a**, **b**) and TEM (**c**, **d**) images of the TiO_2_/PC interlayer

Figure 3 shows the SEM and TEM results for TiO_2_/PC. Figure 3a–c clearly show the uniform ordered porous structure of TiO_2_/PC with a pore size of ~ 110 nm in diameter. The TiO_2_ nanoparticles were evenly distributed in the PC. Figure 3 d shows a lattice spacing of 0.35 nm which corresponds to the (101) facet of anatase TiO_2_ and further illustrates the TiO_2_ nanoparticles were uniformly dispersed in the PC.

**Fig. 4:**
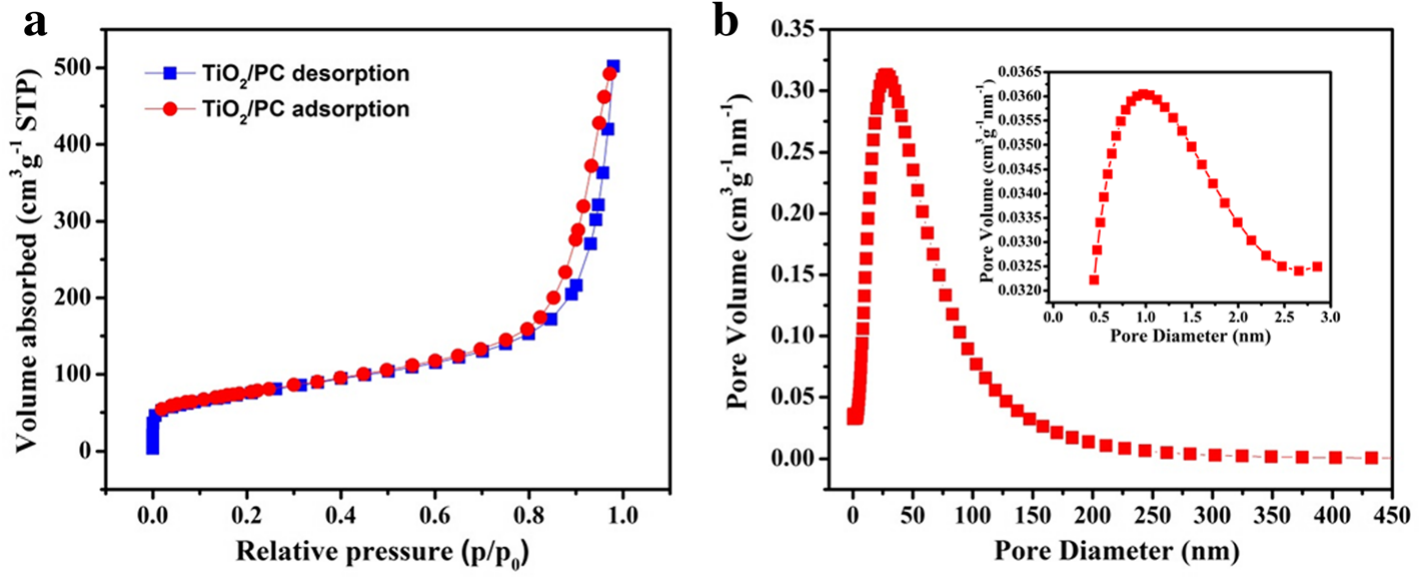
**a** N_2_ adsorption–desorption isotherms. **b** Pore diameter distribution of TiO_2_/PC. Inset: magnification of pore diameter distribution between 0 and 3 nm

Figure 4a shows the nitrogen adsorption–desorption isotherms of the TiO_2_/PC with a BET surface area of 263 m^2^ g^−1^. The pore diameter distribution curve shows the as-prepared TiO_2_/PC composite is composed of small-size micropores around 1 nm (inset) and a relatively broad mesoporous distribution, see Fig. 4b.

**Fig. 5:**
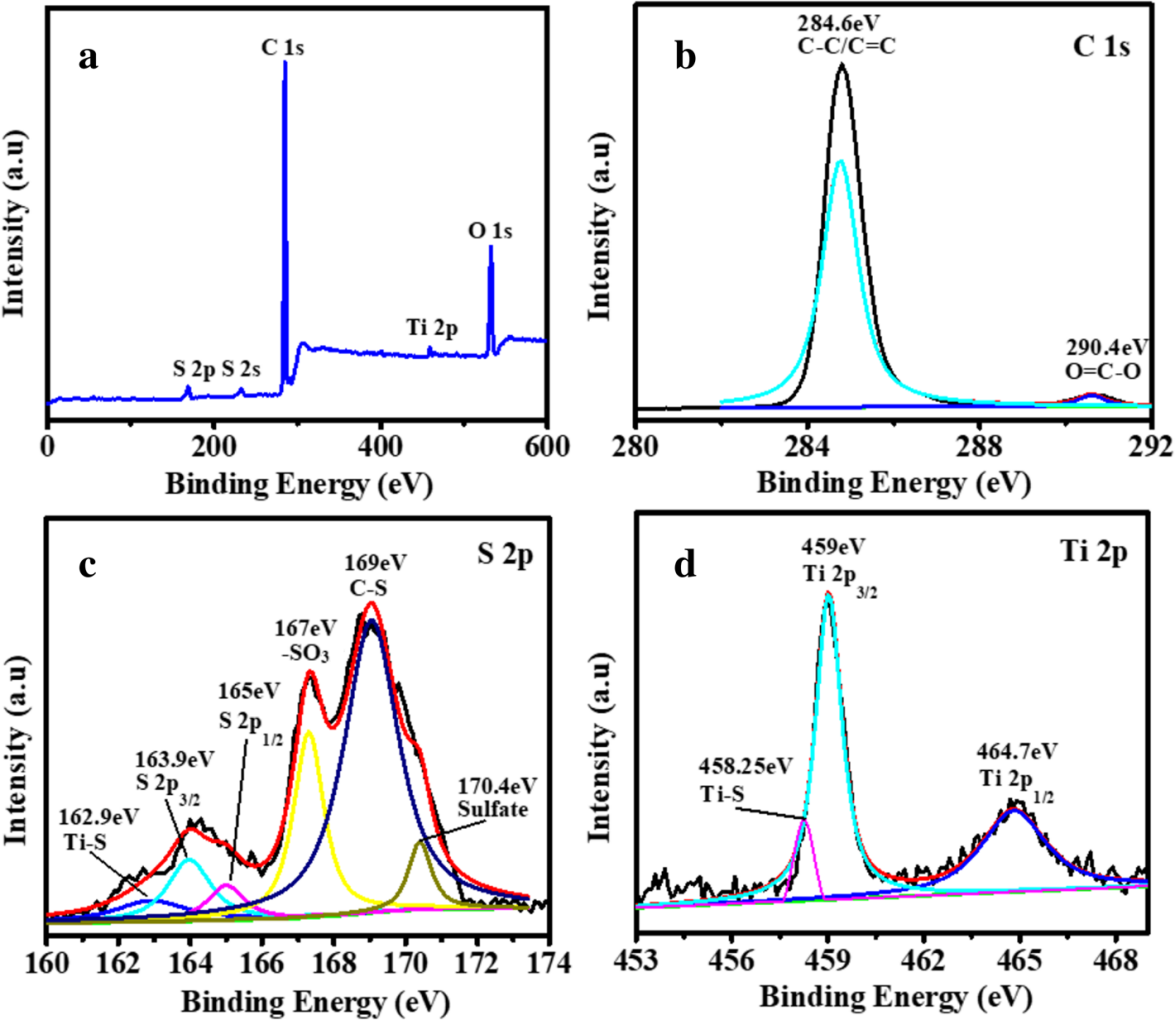
Wide spectrum (**a**) and high-resolution XPS spectra of the TiO_2_/PC-modified separator after charge/discharge spectra of C 1s, S 2p, and Ti 2p (**b**–**d**)

Figure 5a demonstrates the XPS survey spectrum of the TiO_2_/PC-modified separator after charge/discharge, confirming the presence of O, Ti, C, and S in TiO_2_/PC. Figure 5b–d shows the high-resolution XPS spectra of C 1s, S 2p, and Ti 2p. In Fig. 5b, the two peaks in C 1s spectrum can be assigned to two different carbon-containing functional groups, C–C/C=C (284.6 eV) and O–C=O (290.4 eV). In the S 2p spectrum, the weak peak at 162.90 eV corresponds to the S–Ti bond [25, 26], while the three weak peaks at 163.9, 165.0, and 170.40 eV correspond to S 2p_2/3_, S 2p_1/2_, and the sulfate, respectively (Fig. 5c) [27]. The strong peaks located at 167.0 and 169.0 eV correspond to the –SO_3_ and C–S bonds, respectively [28, 29]. The three peaks found in Fig. 5d at 458.25, 459, and 464.7 eV represent Ti–S, Ti 2p_2/3_, and Ti 2p_1/2_, respectively. The presence of a Ti–S bond in the high-resolution XPS spectra of Ti 2p and S 2p reveals the presence of a chemical bond between the elemental sulfur and TiO_2_.

**Fig. 6:**
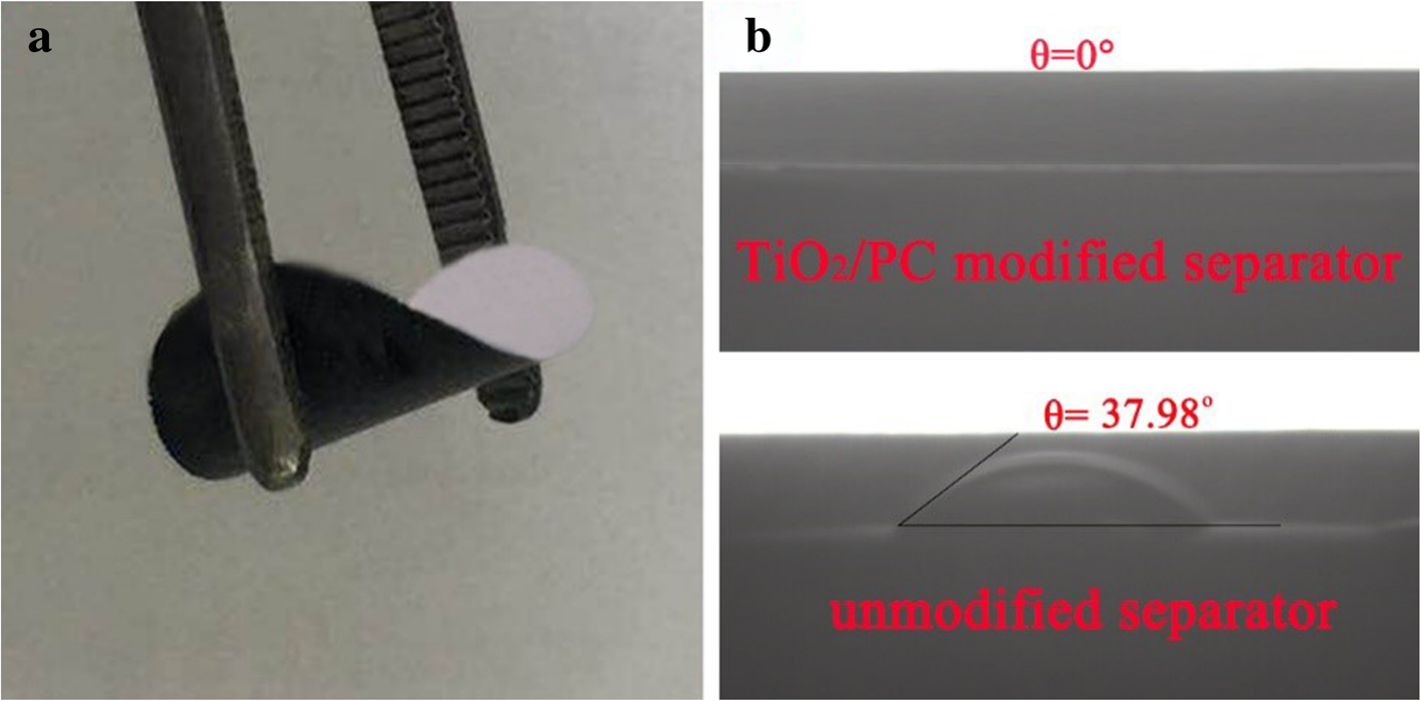
Digital images of the TiO_2_/PC-modified separator with excellent flexibility. (**a**) The contact angle of the electrolyte on the surface of the TiO_2_/PC-modified separator and the unmodified separator (**b**)

Figure 6 a shows the excellent flexibility of the TiO_2_/PC-modified separator. Contact angle measurement was employed to examine the infiltration ability of the electrolyte solution through the TiO_2_/PC-modified separator. Figure 6b shows the contact angle of the electrolyte on the surface of the unmodified separator was 37.98°, while for the TiO_2_/PC-modified separator, it was 0°. This result implies that the TiO_2_/PC coating on the separator improved the electrolyte infiltration due to the polar nature of porous TiO_2_/PC composite.

**Fig. 7:**
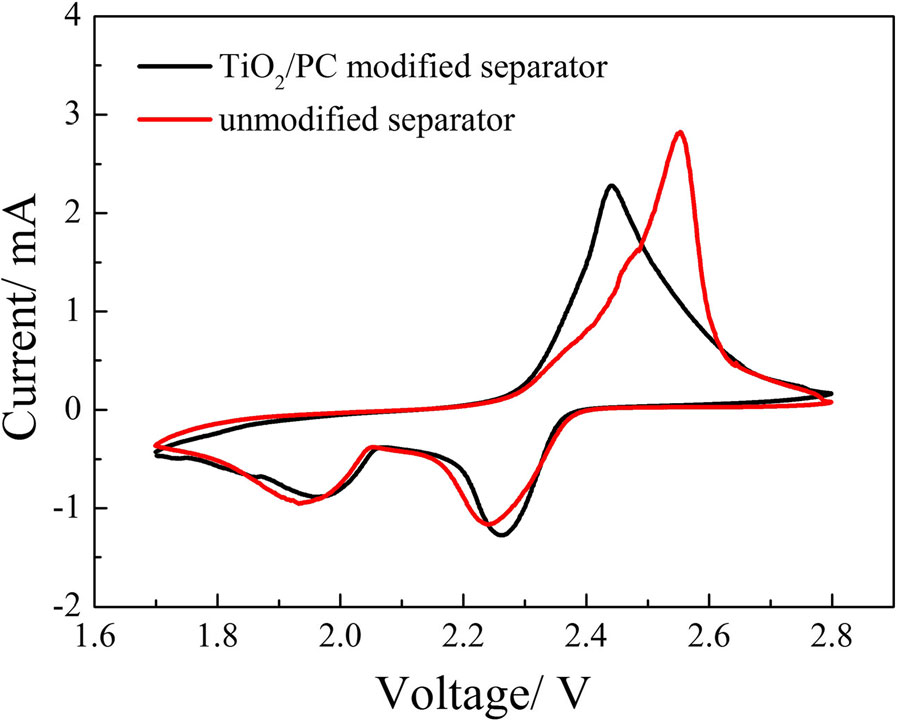
CV curves of the cells with and without TiO_2_/PC-modified separator

The cyclic voltammetry (CV) curves of the Li/S batteries with and without TiO_2_/PC-modified separators were measured at a scan rate of 0.1 mV s^−1^. Both the Li/S batteries exhibit two main cathodic peaks and one anodic peak in Fig. 7. The Li/S battery with TiO_2_/PC-modified separator presents a higher potential cathodic peak at 2.27 V and a relatively lower potential cathodic peak at 1.97 V, corresponding to the reduction of sulfur to soluble polysulfides (Li_2_S_*n*_, 4 ≤ *n* ≤ 8) and then further reduction to Li_2_S/Li_2_S_2_, respectively. The major anodic peak at 2.44 V is ascribed to the conversion of Li_2_S/Li_2_S_2_ to sulfur. Compared to the Li/S battery with pristine separator, the Li/S battery with TiO_2_/PC-modified separator delivers the higher potential cathodic peaks and the smaller potential anodic peak, which indicates that the TiO_2_/PC-modified separator effectively suppresses the potential polarization and enhances the electrochemical kinetics of Li/S batteries.

**Fig. 8:**
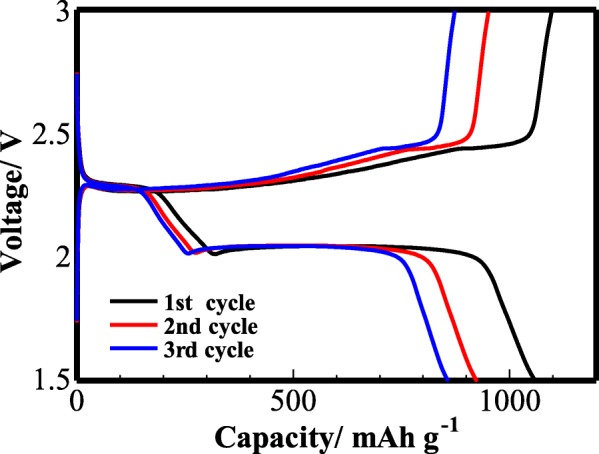
The charge/discharge curves of the cell with TiO_2_/PC-modified Celgard 2400 separator at 0.1 C

The galvanostatic charge/discharge curves for the Li–S cell with TiO_2_/PC-modified Celgard 2400 separator measured at 0.1 C were shown in Fig. 8. Two typical discharge plateaus were observed at 2.27 and 1.97 V, which can be ascribed to the two-step reaction between S and Li. The first plateau can be ascribed to the reduction of the S_8_ and the formation of S_8_^2−^, and the second plateau is related to the reaction of Li_2_S_*n*_, (4 ≤ *n* ≤ 8) to Li_2_S_2_ and Li_2_S [30, 31]. The plateaus during the initial three charge/discharge cycles were presented. The initial discharge capacity was 1060 mAh g^−1^ at 0.1 C. In the second and third cycles, the reversible capacities of 926 mAh g^−1^ and 853 mAh g^−1^, respectively, were achieved, suggesting a good cyclability of the Li–S cell.

**Fig. 9:**
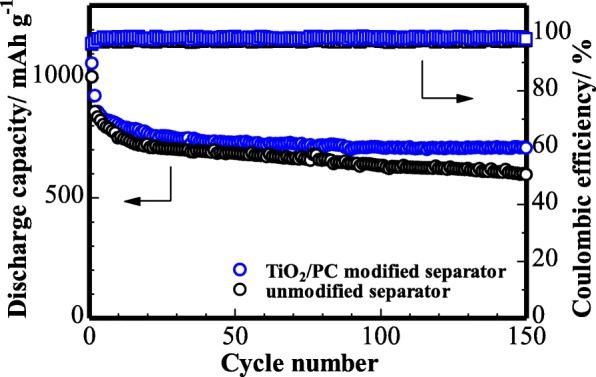
Cycling stability of the cell (with TiO_2_/PC-modified separator and unmodified) at 0.1 C

**Fig. 10:**
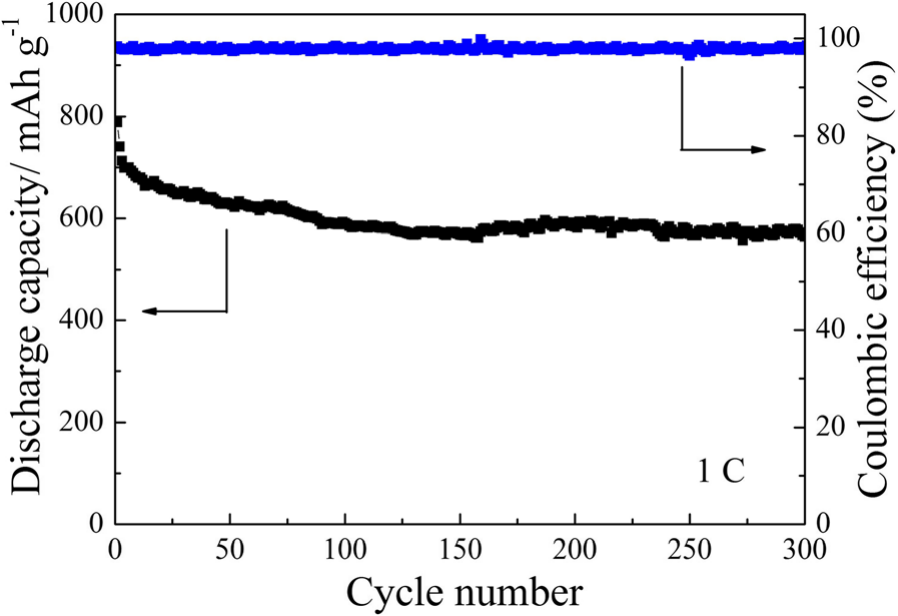
Long-term cycling stability of the cell with TiO_2_/PC-modified separator at 1 C

The cycling performance of the cell with TiO_2_/PC-modified Celgard 2400 separator was investigated. Figure 9 shows that, at 0.1 C, the cell delivers an initial capacity of 1060 mAh g^−1^ and an reversible capacity of 926 mAh g^−1^. After 150 cycles, the battery remains at ~ 75% of the initial reversible capacity (708 mAh g^−1^). On the other hand, the cell with unmodified Celgard 2400 separator shows a lower discharge capacity and a poor cycling performance, indicating that the TiO_2_/PC-modified separator could effectively absorb polysulfides and suppress the shuttle effect. The prolonged cycling life of the cell with TiO_2_/PC-modified Celgard 2400 separator was measured at 1 C (Fig. 10). It delivers an initial discharge capacity of 788 mAh g^−1^ and remains a very stable stability with a reversible capacity of 564 mAh g^−1^ after 300 cycles, which delivers a superior electrochemical performance.

**Fig. 11:**
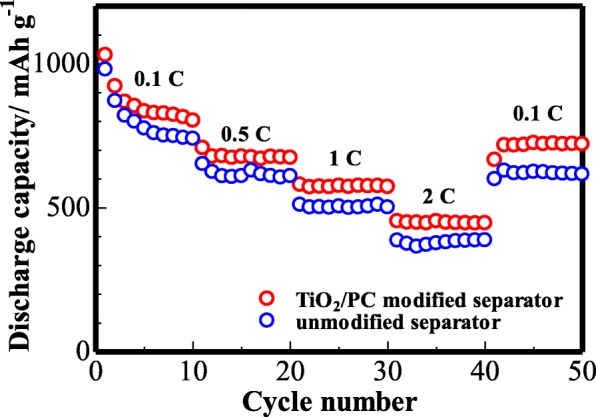
The rate performance of the cell (with unmodified and TiO_2_/PC-modified Celgard 2400 separator) at various current densities

To further investigate the rate capability of the modified cell, a rate performance test was performed (Fig. 11). One can see that the battery with modified Celgard 2400 separator shows reversible capacities of around 823, 672, 578, and 455 mAh g^−1^ at the rate of 0.1, 0.5, 1, and 2 C, respectively. Meanwhile, the discharge capacity could recover to 728 mAh g^−1^ at 0.1 C and remained at ~ 88% of the initial reversible capacity after high-rate cycling, revealing a good capacity recovery. Nevertheless, the battery with unmodified separator exhibits a lower capacity at different current rates. The results further demonstrate that the cell with TiO_2_/PC-modified separator can enhance S utilization and inhibit the polysulfide’s diffusion.

**Fig. 12:**
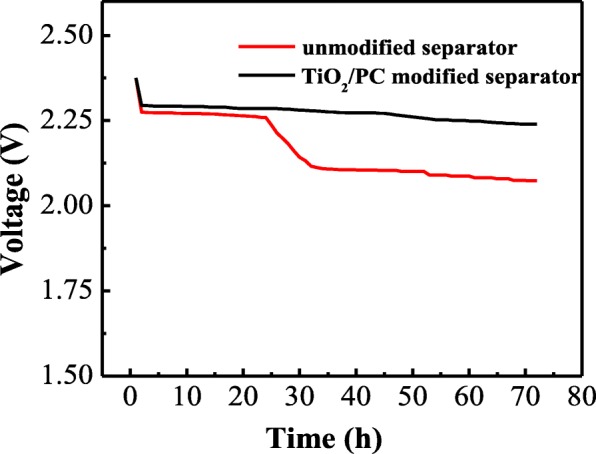
Open-circuit voltage profiles of the cells with unmodified and TiO_2_/PC-modified separator during 72 h rest time

The polysulfide’s diffusion in electrolyte solution results in the self-discharge behavior of the cells. The Li–S batteries with modified and unmodified separator were left to stand (72 h) after the initial 3 cycles at 0.1 C and then tested for further charge/discharge. Figure 12 shows the open-circuit voltage curve for the battery with unmodified separator. It displays an obvious voltage decrease of 0.21 V (2.28~2.07 V) during the rest time, indicating a serious self-reduction process from high-order to low-order polysulfides [32]. Nevertheless, the self-discharge voltage of the cell with TiO_2_/PC-modified separator exhibits only 2.6% decrease of the original open-circuit voltage (2.3~2.24 V) during the rest time, demonstrating that the TiO_2_/PC-modified separator can effectively alleviate the self-discharge of Li–S cell.

## Conclusions

In summary, a TiO_2_/PC-modified Celgard 2400 separator was successfully synthesized for Li/S battery, which can effectively enhance the electrochemical properties of the battery. TiO_2_ could suppress the shuttle effect via electrostatic attraction (S–Ti–O). Meanwhile, the PC in the composite not only enhances the electrical conductivity of the separator, but also inhibits the polysulfide’s diffusion by providing a physical confinement effect within its ordered porous structure. As a result, a high initial specific capacity of 926 mAh g^−1^ is achieved, together with a good cycling stability over 150 cycles. This work provides an effective approach for separator modification for high-performance Li/S batteries.

## Additional File

Additional file 1: **Figure S1.** The cross-section SEM image of TiO_2_/PC on Celgard 2400 separator. **Figure S2.** The cycled SEM image of the TiO_2_/PC modified separator. (DOC 607 kb)

## Abbreviations

DME: 1,2-Dimethoxyethane
DOL: 1,3-Dioxolane
Li/S: Lithium/sulfur
LiTFSI: Lithium bis (trifluoromethanesulfonyl)imide
NMP: *N*-methyl pyrrolidone
PC: Porous carbon
PVDF: Polyvinylidene fluoride
SEM: Scanning electron microscope
TEM: Transmission electron microscope
TEOS: Hydrolyzing tetraethyl orthosilicate
TiO_2_: Titanium dioxide
TTIP: Tetraisopropyl titanate
XPS: X-ray photoelectron spectroscopy
XRD: X-ray diffraction
