# Supplementary material for: TiO2/Porous Carbon Composite-Decorated Separators for Lithium/Sulfur Battery
Source: Nanoscale Res Lett. 2019 May 28;14:176. doi: 10.1186/s11671-019-3010-2 (PMC6538749; doi:10.1186/s11671-019-3010-2)
Supplement: Supplementary file 1 — Figure S1. The cross-section SEM image of TiO2/PC on Celgard 2400 separator. Figure S2. The cycled SEM image of the TiO2/PC modified separator. (DOC 607 kb) [file 11671_2019_3010_MOESM1_ESM.doc]

Additional file 1


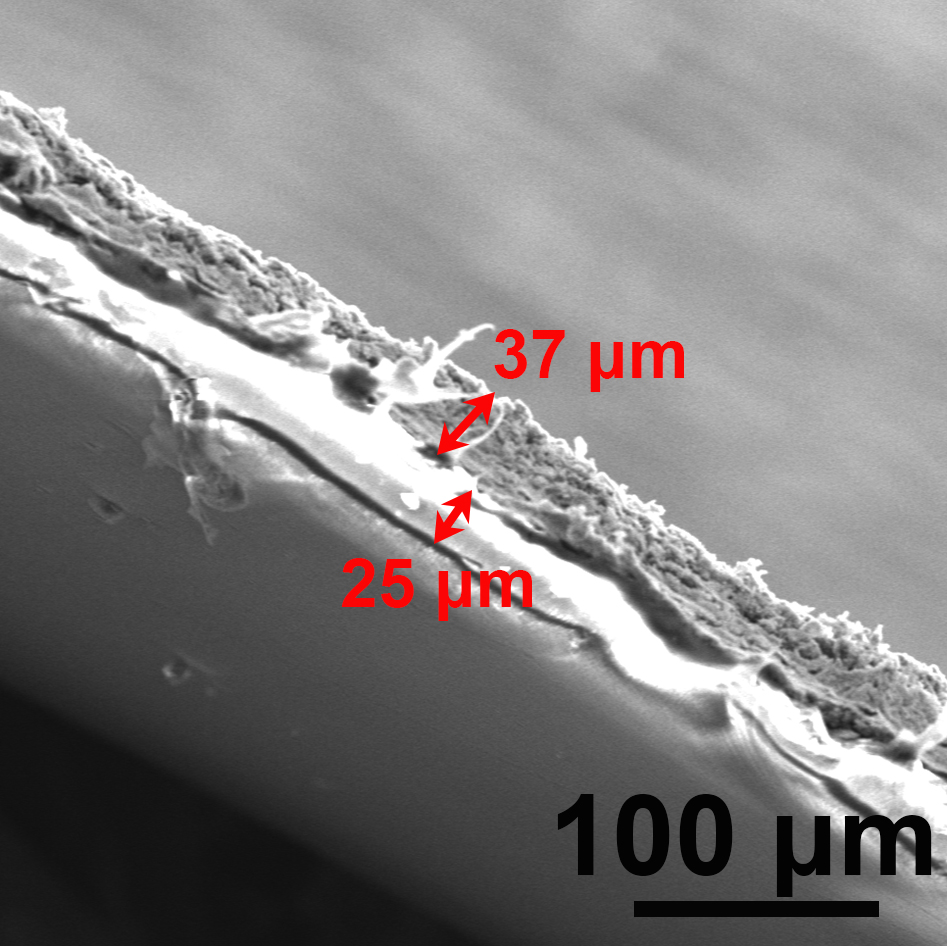


**Figure S1.** The cross-section SEM image of TiO_2_/PC on Celgard 2400 separator


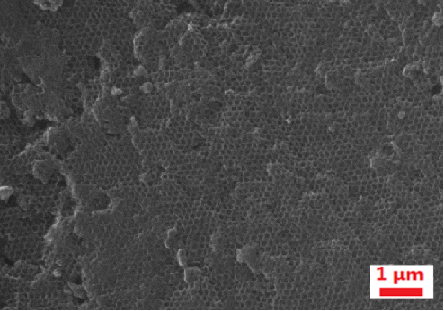


**Figure S2.** The cycled SEM image of the TiO_2_/PC modified separator
